# *CD40* Gene Polymorphisms Associated with Susceptibility and Coronary Artery Lesions of Kawasaki Disease in the Taiwanese Population

**DOI:** 10.1100/2012/520865

**Published:** 2012-05-02

**Authors:** Ho-Chang Kuo, Mei-Chyn Chao, Yu-Wen Hsu, Ying-Chi Lin, Ying-Hsien Huang, Hong-Ren Yu, Ming-Feng Hou, Chi-Di Liang, Kuender D. Yang, Wei-Chiao Chang, Chih-Lu Wang

**Affiliations:** ^1^Department of Pediatrics, Kaohsiung Chang Gung Memorial Hospital and Chang Gung University College of Medicine, Kaohsiung, Taiwan; ^2^Department of Pediatrics, Kaohsiung Medical University Hospital, Kaohsiung, Taiwan; ^3^School of Pharmacy, Kaohsiung Medical University, Kaohsiung, Taiwan; ^4^Department of Medical Genetics, College of Medicine, Kaohsiung Medical University, Kaohsiung, Taiwan; ^5^Cancer Center, Kaohsiung Medical University Hospital, Kaohsiung, Taiwan; ^6^Institute of Clinical Medicine, College of Medicine, Kaohsiung Medical University, Kaohsiung, Taiwan; ^7^Departments of Medical Research and Pediatrics, Show Chwan Memorial Hospital in Chang Bing, Changhua, Taiwan; ^8^Department of Pediatrics, Po-Jen Hospital, Kaohsiung, Taiwan

## Abstract

*Background*. Kawasaki disease (KD) is characterized by systemic vasculitis of unknown etiology. Our previous studies showed expression of *CD40* ligand on CD4+ T cells correlated to the coronary artery lesion (CAL) and disease progress in KD. Other studies from Japan suggested the role of *CD40L* in the pathogenesis of CAL, and this might help explain the excessive number of males affected with KD but cannot be reproduced by Taiwanese population. This study was conducted to investigate the *CD40* polymorphism in KD and CAL formation. *Methods*. A total of 950 subjects (381 KD patients and 569 controls) were investigated to identify 2 tagging single-nucleotide polymorphisms (tSNPs) of *CD40* (rs4810485 and rs1535045) by using the TaqMan allelic discrimination assay. *Results*. A significant association was noted with regards to *CD40* tSNPs (rs1535045) between controls and KD patients (*P* = 0.0405, dominant model). In KD patients, polymorphisms of *CD40* (rs4810485) showed significant association with CAL formation (*P* = 0.0436, recessive model). Haplotype analysis did not yield more significant results between polymorphisms of *CD40* and susceptibility/disease activity of KD. *Conclusions*. This study showed for the first time that polymorphisms of *CD40* are associated with susceptibility to KD and CAL formation, in the Taiwanese population.

## 1. Introduction

Kawasaki disease (KD) is characterized by acute, febrile, systemic vasculitis and was first described by Kawasaki et al. in 1974 [1]. The most serious complication of KD is the occurrence of coronary artery lesions (CALs) [2]. In developed countries, KD is the leading cause of acquired heart diseases in children. KD primarily affects children less than 5 years of age [3], and the prevalence is highest in Japan, followed by Korea and Taiwan, and lowest in Europe [4]. 

The etiology of KD is still under investigation. Esper et al. reported that human coronavirus is associated with KD but cannot be reproduced by other groups [5]. Previous studies have either failed to identify the causative pathogen for KD or reported discrepant results [5–7]. Evidence suggests that immune activation with vascular endothelial inflammation may be involved in the pathogenesis of KD and CAL formation. Acute stage of KD is reported to be associated with overactivation of immunologic factors including immune-competent cell activation [4], cytokines [8, 9], nitric oxide production [10], autoantibody production [11, 12], and adhesion molecule expression [13, 14]. 

CD40 ligand (CD40L, CD154, gp 39), a transmembrane protein structurally related to tumor necrosis factor-alpha, was originally identified on activated CD4+ T cells. Both membrane-bound and soluble forms of CD40L may interact with CD40, which is mainly expressed on B cells, macrophages, endothelial cells, and vascular smooth muscle cells, resulting in various immune and inflammatory responses [15, 16]. Interaction between CD40L and CD40 plays a central role in the activation of the immune system, such as immunoglobulin G (IgG) switching, autoimmune disease, antiviral effect, allograft rejection, cytokines regulation, arthrosclerosis, and endothelial cell interaction [17, 18]. CD40-CD40L system is also associated with both prothrombotic and proinflammatory effects [19, 20]. The soluble form of CD40L (sCD40L) is derived mainly from activated platelets and contributes to the pathophysiology of atherosclerosis and atherothrombosis [20]. Indeed, sCD40L has autocrine, paracrine, and endocrine activities, and it enhances platelet activation, aggregation, and platelet-leucocyte conjugation that may lead to atherothrombosis. It also has been suggested that sCD40L may play a pathogenic role in triggering acute coronary syndromes [19]. By detecting expression level of CD40L from clinical samples, our previous study provides evidence that supports a functional role of CD40L in the susceptibility of KD and CAL formation [21]. 

Several reports have indicated that genetic polymorphisms may contribute to the susceptibility and disease severity of KD. For example, polymorphisms in the genes including IL-10, CASP3, IL-18, and inositol 1,4,5-trisphosphate 3-kinase C (*ITPKC*) have been reported to be involved in the development of KD [22–26]. In 2004, Onouchi and colleagues were the first group reporting the association between CD40L gene polymorphisms and KD [27]. Our previous studies have shown that expression of CD40L on CD4+ T cells was correlated to the coronary artery lesion (CAL) and disease progress in KD [21]. However, another group from Taiwan reported the lack of an association between CD40L polymorphisms and susceptibility KD in a Taiwanese population [28]. Therefore, we strived to investigate the potential genetic role of CD40 in the susceptibility of KD and CAL formation in the Taiwanese population.

## 2. Material and Methods

### 2.1. Patients Studied

All subjects studied were children who filled the diagnostic criteria for KD according to 2004 AHA criteria [29] and were admitted to the Kaohsiung Chang Gung Memorial Hospital between 2000 and 2010. All patients were treated with a single infusion of IVIG (2 g/kg) administered over a 12-hour period. Aspirin was administered until all signs of inflammation were resolved or regression of CAL was detected under two-dimensional (2D) echocardiography as in our previous reports [30–32]. This study was approved by the Institutional Review Board of the Chang Gung Memorial Hospital. CAL was defined by the internal diameter of the coronary artery being at least 3 mm (or 4 mm if the subject was over the age of 5 years) or the internal diameter of a segment being at least 1.5 times of an adjacent segment in the echocardiogram [22, 33]. IVIG responsiveness was defined as defervescence within 48 hours after the completion of IVIG treatment and being without fever (temperature, >38°C) [2, 34].

### 2.2. DNA Extraction

Blood cells were subjected to DNA extraction by treating them first with 0.5% SDS lysis buffer and then protease K (1 mg/mL) for digestion of nuclear protein for 4 h at 60°C. Using the Gentra extraction kit followed by 70% alcohol precipitation, total DNA was harvested. 

### 2.3. Genotyping

Two tagging SNPs (rs4810485 and rs1535045) in the *CD40* gene with a minor allele frequency of >10% in the Han Chinese population were selected from the HapMap database (http://www.hapmap.org/). Genotyping was performed by using the TaqMan allelic discrimination assay (Applied Biosystems, Foster city, CA, USA) was noted in our previous report [22, 24, 35]. Briefly, PCR primers and TaqMan minor groove binding probes were designed by applied Biosystems. The polymerase chain reaction (PCR) was carried out in a 96-well microplate with the ABI 9700 Thermal Cycler. After PCR, fluorescence was detected by ABI 7500 Real-Time PCR system and data was analyzed using the System SDS software (version 1.2.3).

### 2.4. Statistical Analysis

JMP 9.0 for Windows was used for analysis. The statistical differences between case and control in genotype and allele frequency were assessed by the *χ* ^2^-test. Statistical differences in genotype and allele frequency of KD patients with/without CAL formation were assessed using the *χ* ^2^ test. Linkage disequilibrium (LD) was assessed for one pair of SNPs, and haplotype blocks were defined using the default setting of the Haploview software 4.1 (Broad Institute, Cambridge, MA, USA).

## 3. Results

### 3.1. A Significant Association between Genetic Polymorphisms in CD40 and Susceptibility to Kawasaki Disease

A total of 950 subjects including 381 KD patients and 569 controls were recruited in this study.  Table 1 shows the characteristics of the subjects. The average age of cases was 1.7 years and that in the controls is 5.7 years. CAL was observed in 9.7% (37/381) of KD patients 8 weeks after disease onset, and 12.9% (49/381) of the KD patients were resistant to initial IVIG treatment. The genotype frequencies of the controls and patients were in the Hardy-Weinberg equilibrium (Table 2). Polymorphism rs1535045 in the CD40 was found to have significant influence with regards to the susceptibility of KD (*P* = 0.0405). 

### 3.2. CD40 Genetic Polymorphisms Associated with CAL Formation in the KD Patients

The association between CD40 genotypes and CAL formation, the major complication of KD, was evaluated. 37 patients among the 381 KD patients developed CAL. Our results indicated that the TT genotype of rs4810485 has protective effects for CAL formation in KD patients (Table 3).

### 3.3. Haplotype Analysis of *CD40* in the KD Patients

We further calculated pairwise linkage disequilibrium (LD) and analyzed haplotypes of *CD40*. However, no significant association was found between CD40 haplotype analysis and the susceptibility of KD (Table 4), as well as CAL formation (Table 5).

## 4. Discussion

Kawasaki disease is caused by activation of the immune system targeting on vascular endothelium, resulting in systemic vasculitis or even coronary artery lesions formation. Interaction of CD40L with its receptor CD40 has been implicated in the modulation of immune and inflammatory responses, which are critical for the activation of tissue structure cells, such as endothelial cells, smooth muscle cells, epithelial cells, and fibroblast and induce production of a cascade of proinflammatory cytokines [18, 19]. The CD40-CD40L signaling pathway has been associated with the pathogenic processes of chronic inflammatory diseases, including autoimmune diseases, neurodegenerative disorders, graft-versus-host disease, cancer, vasculitis, and atherosclerosis [17, 20]. In the presented study, our results implied that genetic polymorphisms in CD40 may result in the overactivation of proinflammatory signaling pathways that lead to the development of KD. 

Our previous report indicated that expression of CD40L on CD4+ T-cells correlated to the CAL formation and disease progress in KD patients [21]. Burns and Glodé also suggested that CD40-CD40L interaction impacts on the vasculitis pathogenesis of KD [4]. Therefore, the CD40-CD40L signaling pathway may play an important pathogenic role in acute coronary syndromes such as the CAL of patients with KD. In this study, we found that CD40 genetic polymorphism (rs1535045) is associated with susceptibility of KD. Interestingly, rs1535045 in *CD40* gene has been confirmed to be associated with the coronary artery calcification in diabetic families [36]. These observations in combination with those of the present study support genetic polymorphism effects of CD40 in the vascular diseases. Further study into the mechanisms between CD40-CD40L interaction and long-term coronary arterial vasculitis may provide a better understanding for the pathogenesis of KD. 

Although our studies indicated the significant association between genetic polymorphisms of CD40 and the risk of KD, haplotypes of KD didn not yield significant results. We acknowledged that the modest sample size in the study was underpowered to detect the small genetic effect of CD40 in the disease severity such as CAL formation. These findings need to be replicated in a second population with a larger sample size.

Taken together, our results indicated that the genetic polymorphisms of CD40 are very likely to be involved in the susceptibility and disease severity of KD in a Taiwanese population.

## Figures and Tables

**Table 1:** Basal characteristics of patients with Kawasaki disease and normal controls.

Characteristics	Patients with KD	Normal control
	*N* = 381	*N* = 569
Mean (SD) age (years)	1.7 ± 1.6	5.7 ± 4.9
Age range (years)	0–11	0–51
CAL formation	37 (9.7%)	
IVIG resistance	49 (12.9%)	

CAL: coronary artery lesions; IVIG: intravenous immunoglobulin; SD: standard deviation.

**Table 2:** Genotype and allele frequencies of the *CD40* gene in controls and patients with Kawasaki disease.

	Genotype	Case (%) (*n* = 381)	Control (%) (*n* = 569)	Allele	Case (%) (*n* = 381)	Control (%) (*n* = 569)	Genotype *P* value	Dominant *P* value	Recessive *P* value	Allelic *P* value
rs4810485	TT	50 (13.2)	99 (17.7)	T	284 (37.4)	460 (41.1)	0.1632	0.3489	0.0609	0.1005
	GT	184 (48.4)	262 (46.9)	G	476 (62.6)	658 (58.9)				
	GG	146 (38.4)	198 (35.4)							
rs1535045	TT	44 (11.5)	61 (10.8)	T	262 (34.4)	346 (30.6)	0.1160	0.0405*	0.7180	0.0856
	CT	174 (45.7)	224 (39.6)	C	500 (65.6)	784 (69.4)				
	CC	163 (42.8)	280 (49.6)							

*Significant (*P* < 0.05) values are in bold.

**Table 3:** Genotype and allele frequencies of the *CD40* gene in patients with or without coronary artery lesion (CAL) formation.

	Genotype	CAL (%) (*n* = 37)	Without (%) (*n* = 336)	Allele	CAL (%) (*n* = 37)	Without (%) (*n* = 336)	Genotype *P* value	Dominant *P* value	Recessive *P* value	Allelic *P* value
rs4810485	TT	1 (2.7)	49 (14.6)	T	25 (33.8)	253 (37.8)	0.0666	0.6381	0.0436*	0.5021
	GT	23 (62.2)	155 (46.3)	G	49 (66.2)	417 (62.2)				
	GG	13 (35.1)	131 (39.1)							
rs1535045	TT	2 (5.4)	41 (12.2)	T	23 (31.1)	233 (34.7)	0.4410	0.9641	0.2192	0.5368
	CT	19 (51.4)	151 (44.9)	C	51 (68.9)	439 (65.3)				
	CC	16 (43.2)	144 (42.9)							

*Significant (*P* < 0.05) values are in bold.

**Table 4:** Haplotype frequencies of the *CD40 *gene in controls and patients with Kawasaki disease.

rs4810485/rs1535045	Case (%) (*n* = 381)	Control (%) (*n* = 569)	OR (95% CI)	*P* Value
G/T	258 (34.0)	343 (30.7)	1.22 (0.98–1.52)	0.0767
G/C	216 (28.5)	317 (28.3)	1.10 (0.88–1.39)	0.3937
T/C	282 (37.2)	457 (40.8)	Reference	

Haplotype frequency less than 1% was excluded.

**Table 5:** Haplotype frequencies of the *CD40 *gene in patients with or without coronary artery lesion formation.

rs4810485/rs1535045	CAL (%) (*n* = 37)	Without (%) (*n* = 336)	OR (95% CI)	*P* value
G/T	23 (31.1)	231 (34.5)	1.00 (0.55–1.81)	0.9991
G/C	26 (35.1)	186 (27.8)	1.40 (0.79–2.51)	0.2511
T/C	25 (33.8)	251 (37.5)	Reference	

## Abbreviations

KD: Kawasaki disease
IVIG: Intravenous immunoglobulin
CAL: Coronary artery lesions.
